# Supplementary figures and images for: Coarse-grained model of serial dilution dynamics in synthetic human gut microbiome
Source: PLoS Comput Biol. 2025 Jul 14;21(7):e1013222. doi: 10.1371/journal.pcbi.1013222 (PMC12270328; doi:10.1371/journal.pcbi.1013222)

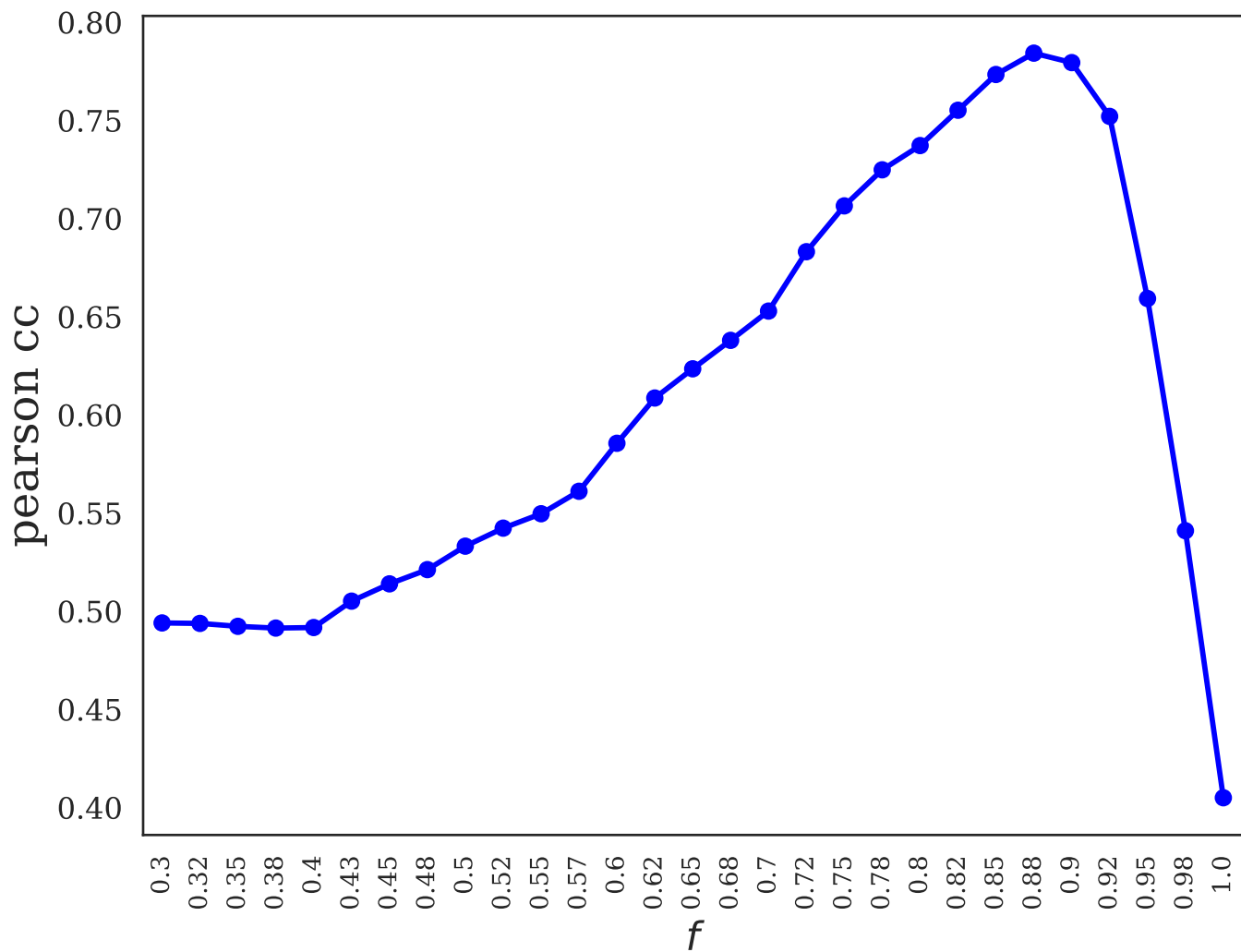

Supplement: S1 Fig — The time fraction fwas estimated by inferring Ri from our model for linearly spaced values of f in the interval [0.3,1]. For each estimated Ri, we made a prediction of the steady-state strain abundances starting from the inoculum using our model. Pearson’s correlation coefficients between predicted and observed abundances for different values of f are plotted. (PDF) [file pcbi.1013222.s001.pdf]

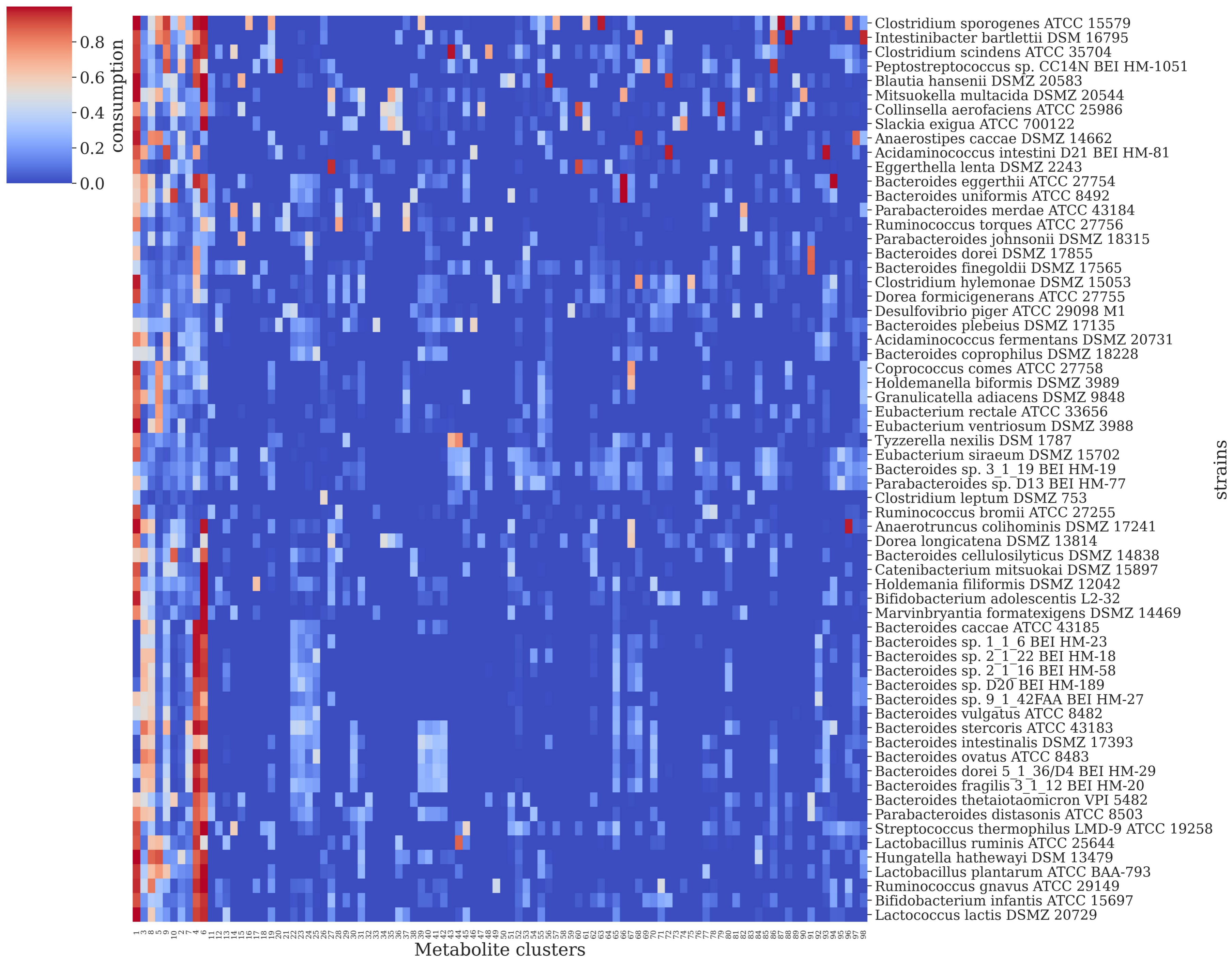

Supplement: S2 Fig — Columns and rows represent the metabolite clusters and the strains, respectively. From the left, the first 10 columns represent the non-singleton clusters, followed by the 88 singletons. (PDF) [file pcbi.1013222.s002.pdf]

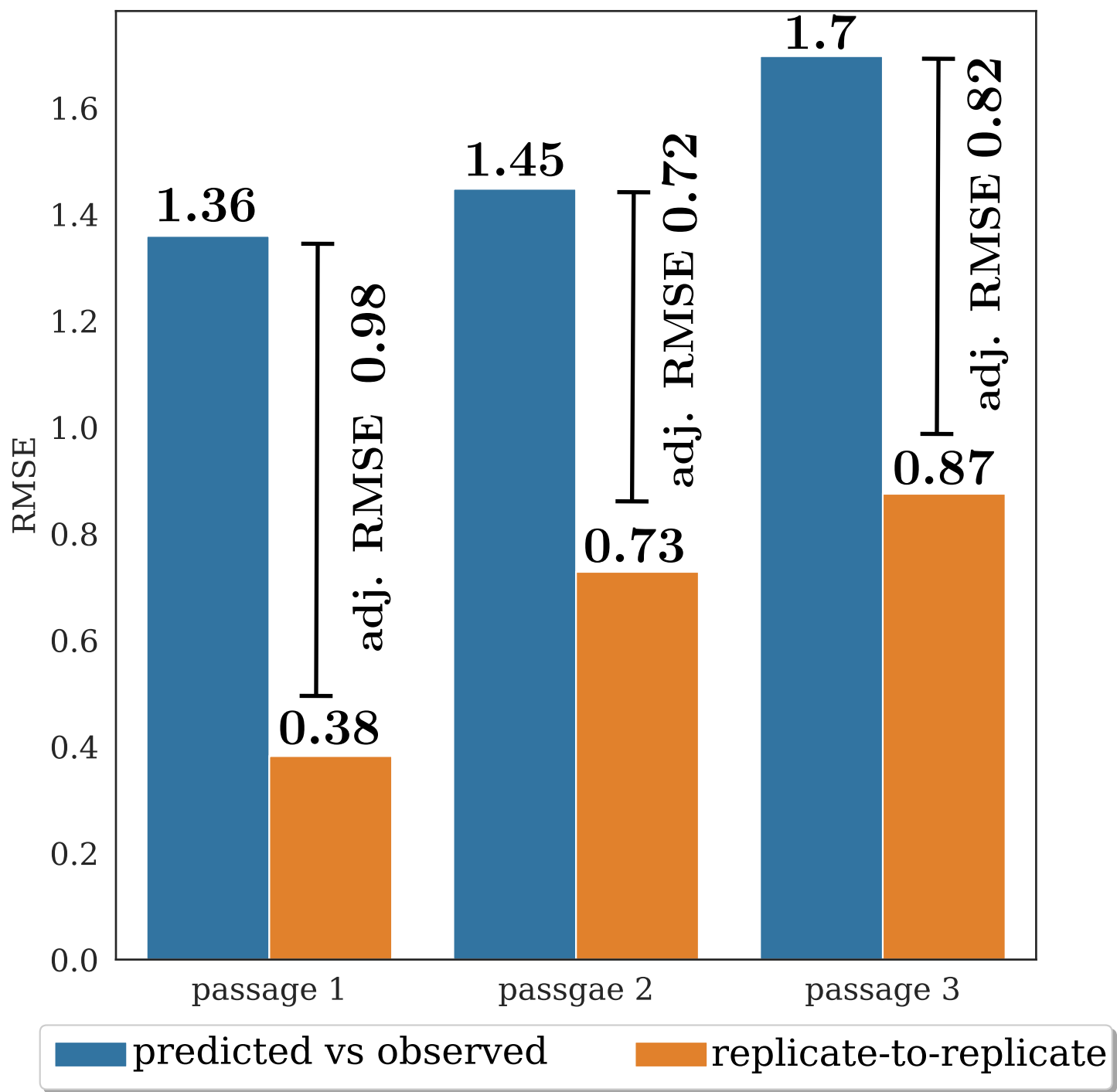

Supplement: S3 Fig — Biological replicate-to-replicate variability at each passage was estimated as the root mean squared error (RMSE) between the strain abundances for the biological replicates. Model performance at each passage was estimated as the RMSE between predicted and observed strain abundances. The adjusted RMSE was then calculated as the difference between the RMSE for model performance and the RMSE for biological replicate-to-replicate variability. (PDF) [file pcbi.1013222.s003.pdf]

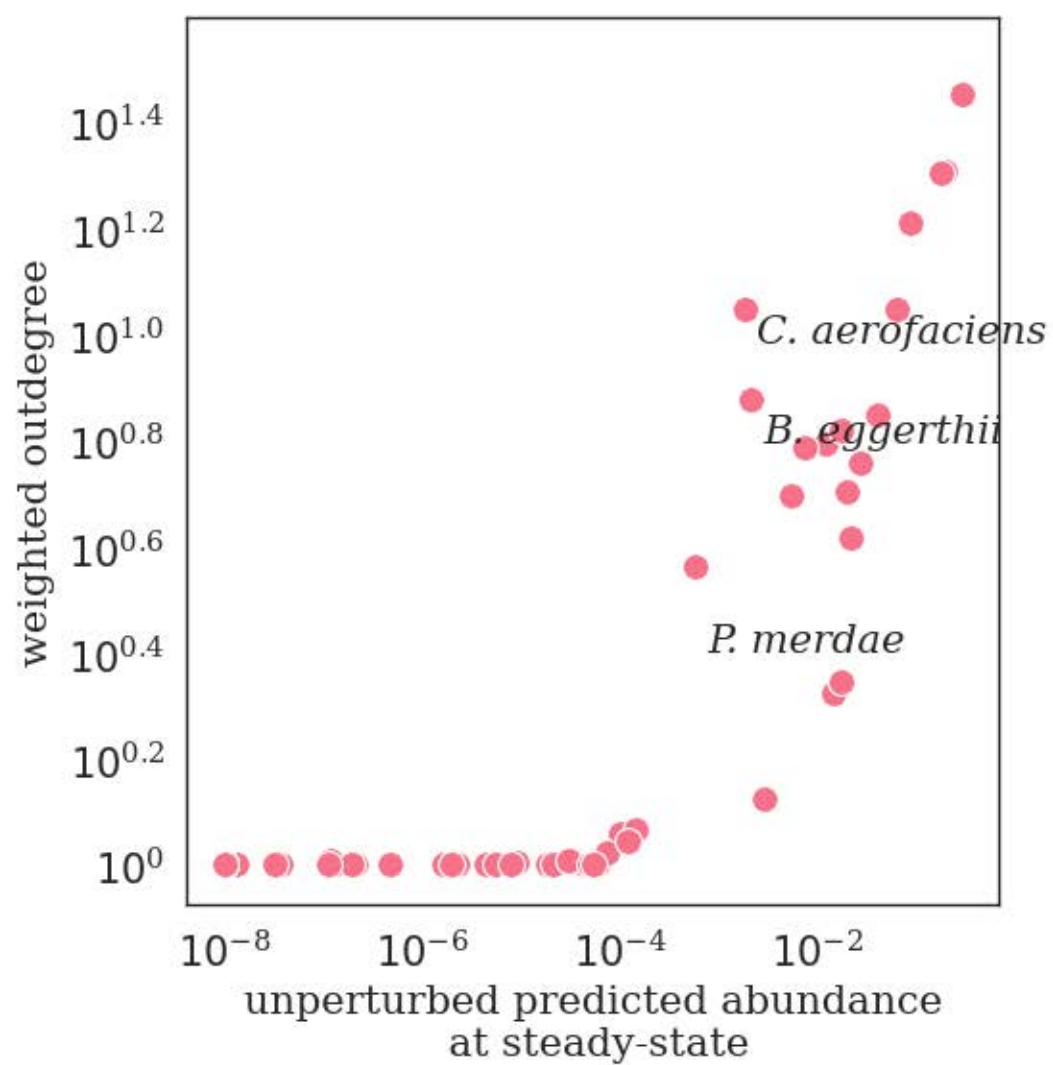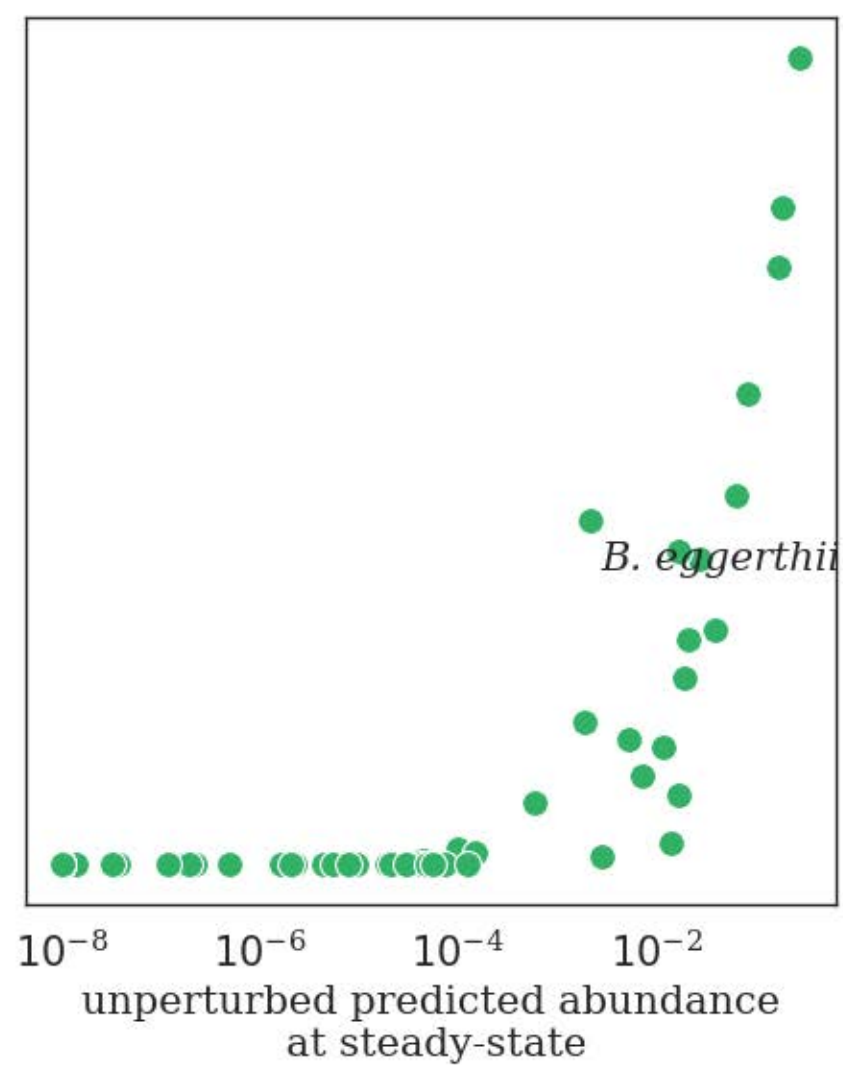

Supplement: S5 Fig — Total weighted out-degree restricted to competitive (red, a)) and cooperative (red, b)) edges in the strain-strain interaction network for all the strains plotted against predicted strain abundances at steady state (passage 3) for the unperturbed hCom2 community. Some intermediate abundance strains have been highlighted in the plots. These strains have a disproportionately large impact on the community compared to their abundances in the unperturbed community. (PDF) [file pcbi.1013222.s005.pdf]

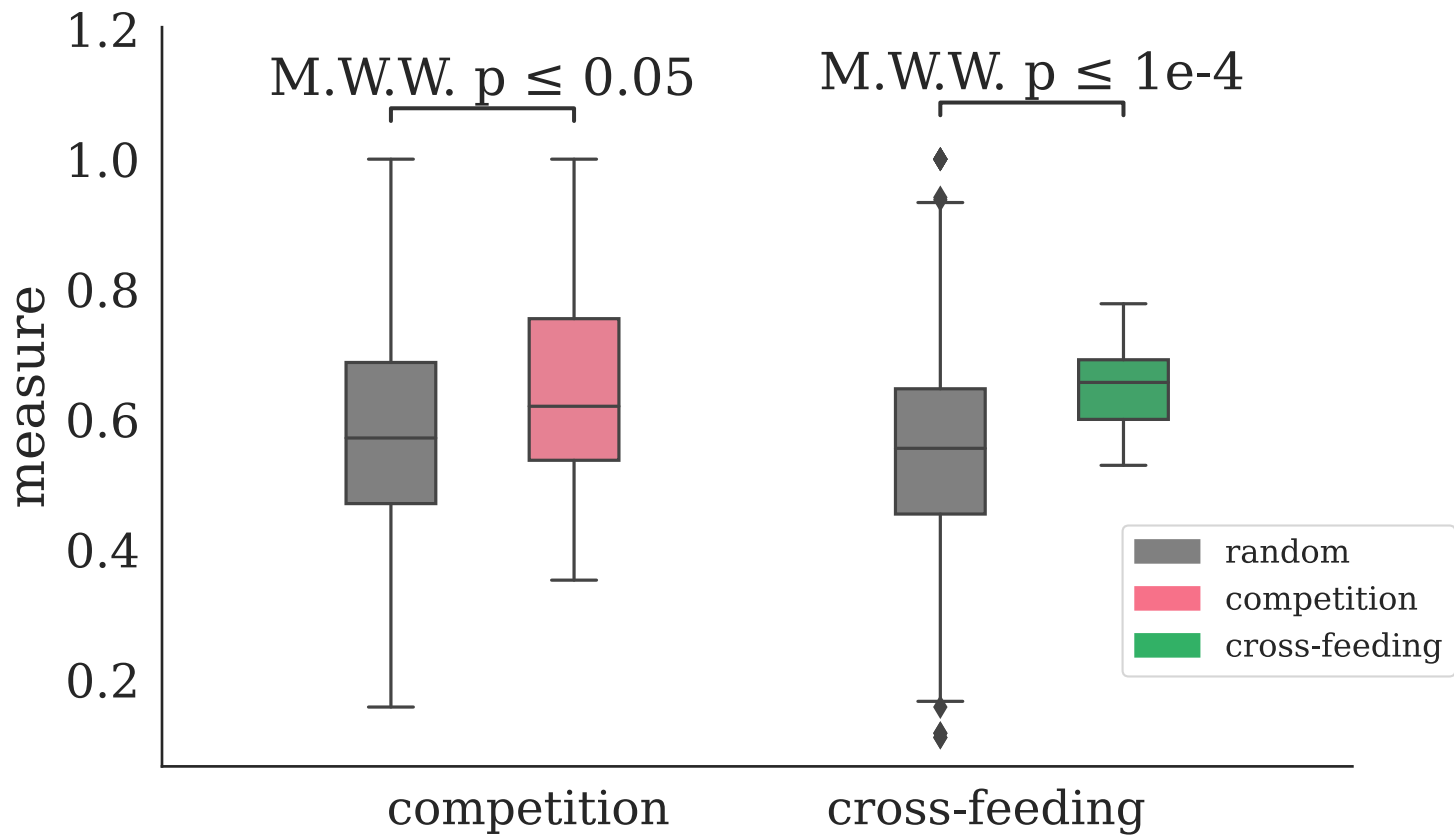

Supplement: S7 Fig — (Left) Comparison of competition scores for competitive edges (red) against random edges. Non-interacting and cooperative edges (green) are defined as random in this context. We obtained a mean competition score of 0.65 for competitive edges vs. 0.58 for non−−interacting or cooperative interaction edges (p=0.02, two-tailed Mann-Whitney Wilcoxon test). (Right) Comparison of cross-feeding scores for cooperative edges (green) against random edges. Non-interacting and competitive edges (red) are defined as random in this context. We obtained a mean cross-feeding score of 0.64 for cooperative edges vs. 0.55 for non−−interacting or competitive interaction edges (p=9.7 × 10^−5^, two-tailed Mann-Whitney Wilcoxon test). (PDF) [file pcbi.1013222.s007.pdf]

average corr. = 0.23,  
p-value =  $7.57 \times 10^{-20}$

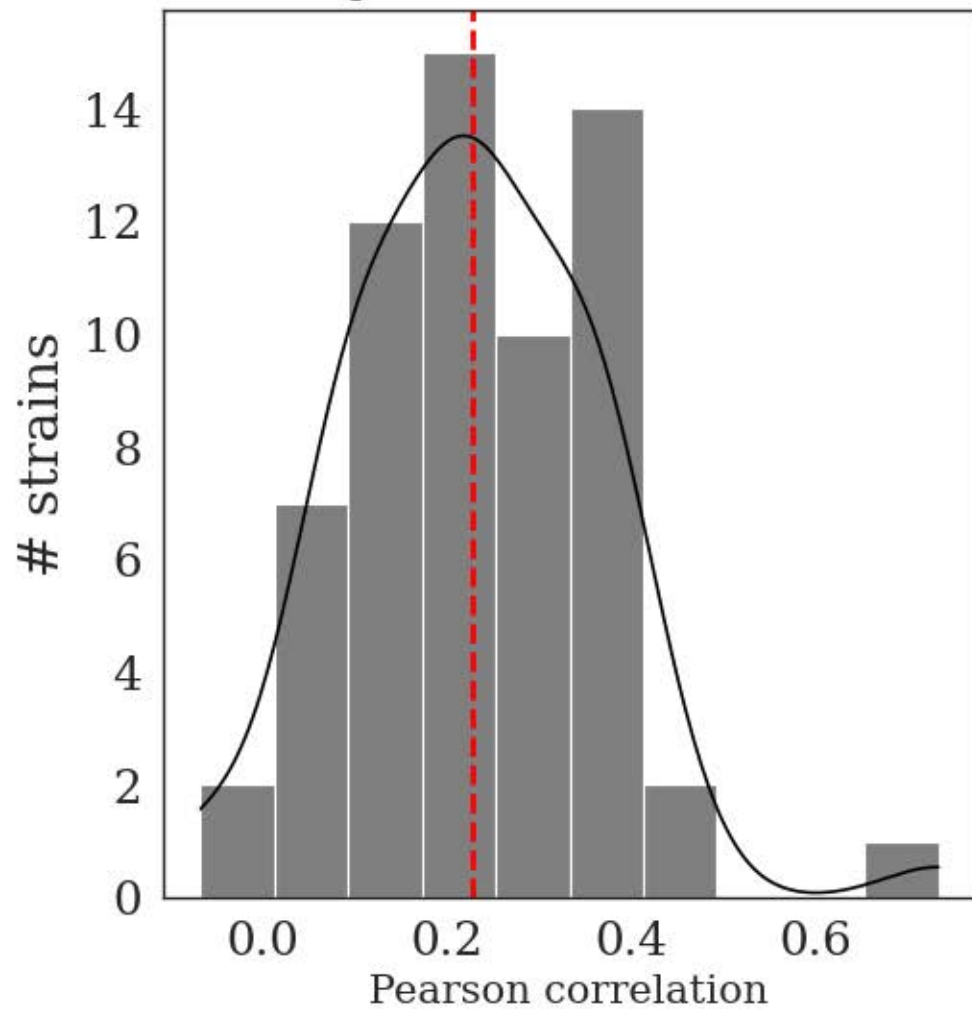

average corr. = 0.27,  
p-value =  $2.62 \times 10^{-27}$

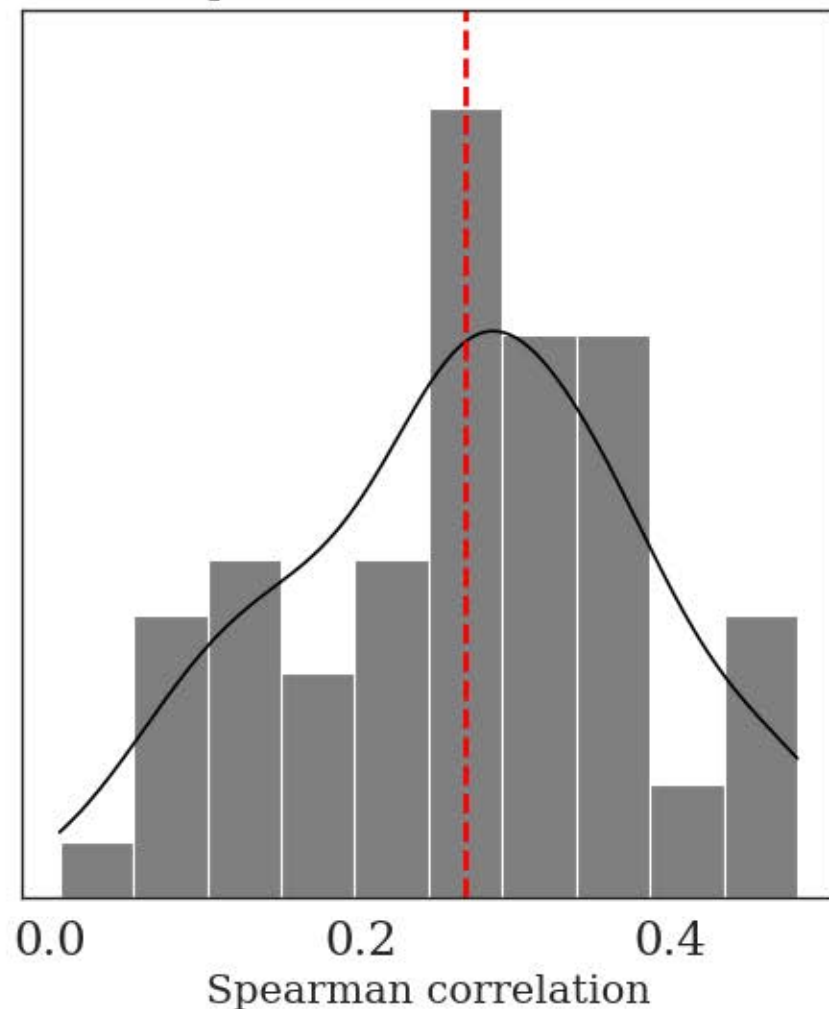

Supplement: S8 Fig — Histogram across strains of a) Pearson’s and b) Spearman’s correlation coefficients between the predicted log-fold change in strain abundances (in response to resource perturbations) and the consumption fluxes. The black solid curves represent the kernel density estimates. The red dashed lines show the average values. (PDF) [file pcbi.1013222.s008.pdf]

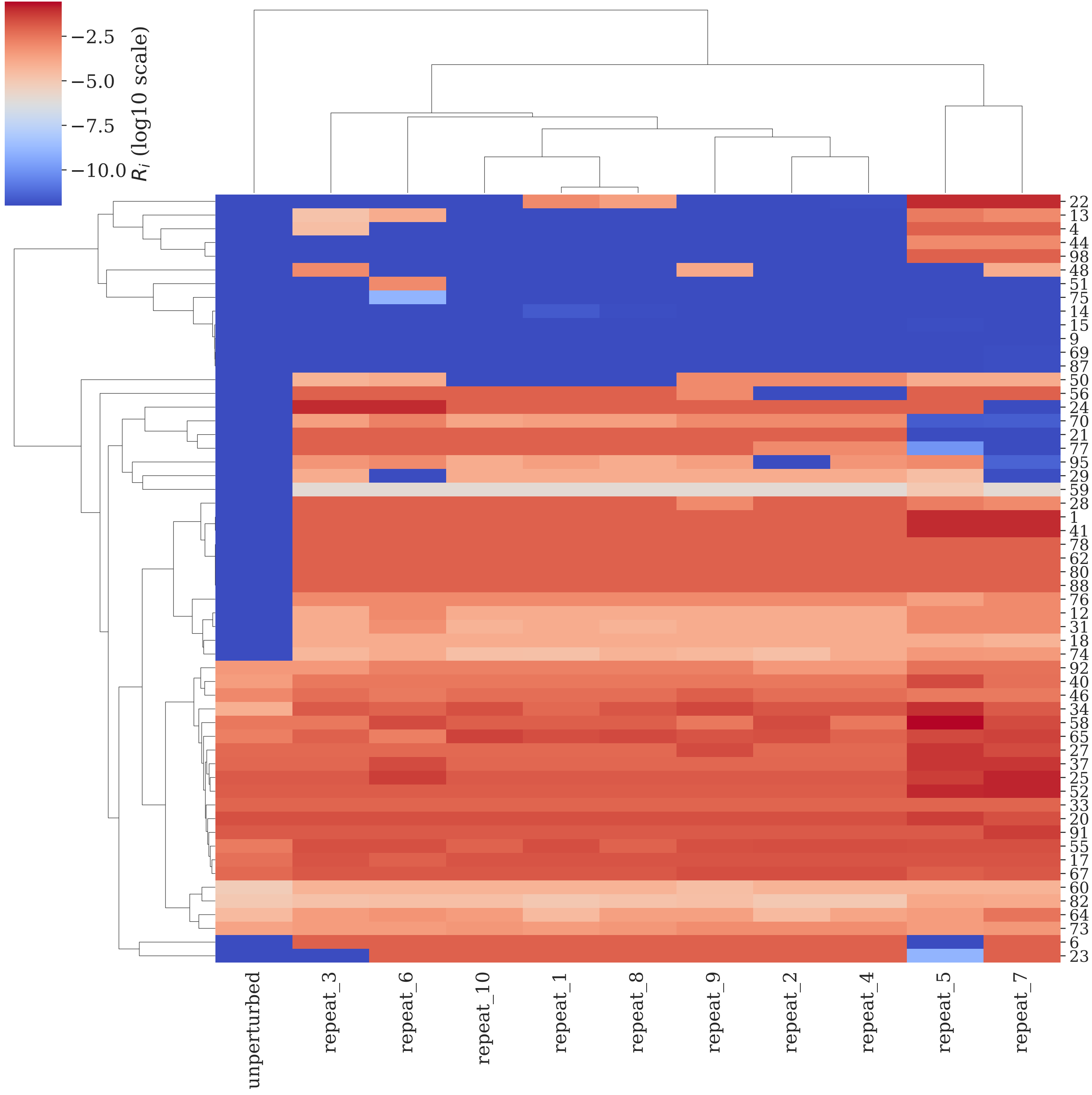

Supplement: S9 Fig — Hierarchically clustered heatmap of the 10 perturbed Ri solutions generated by our greedy algorithm for equalising strain abundances at steady-state for hCom2. The columns and rows represent the different solutions and metabolite clusters, respectively. (PDF) [file pcbi.1013222.s009.pdf]

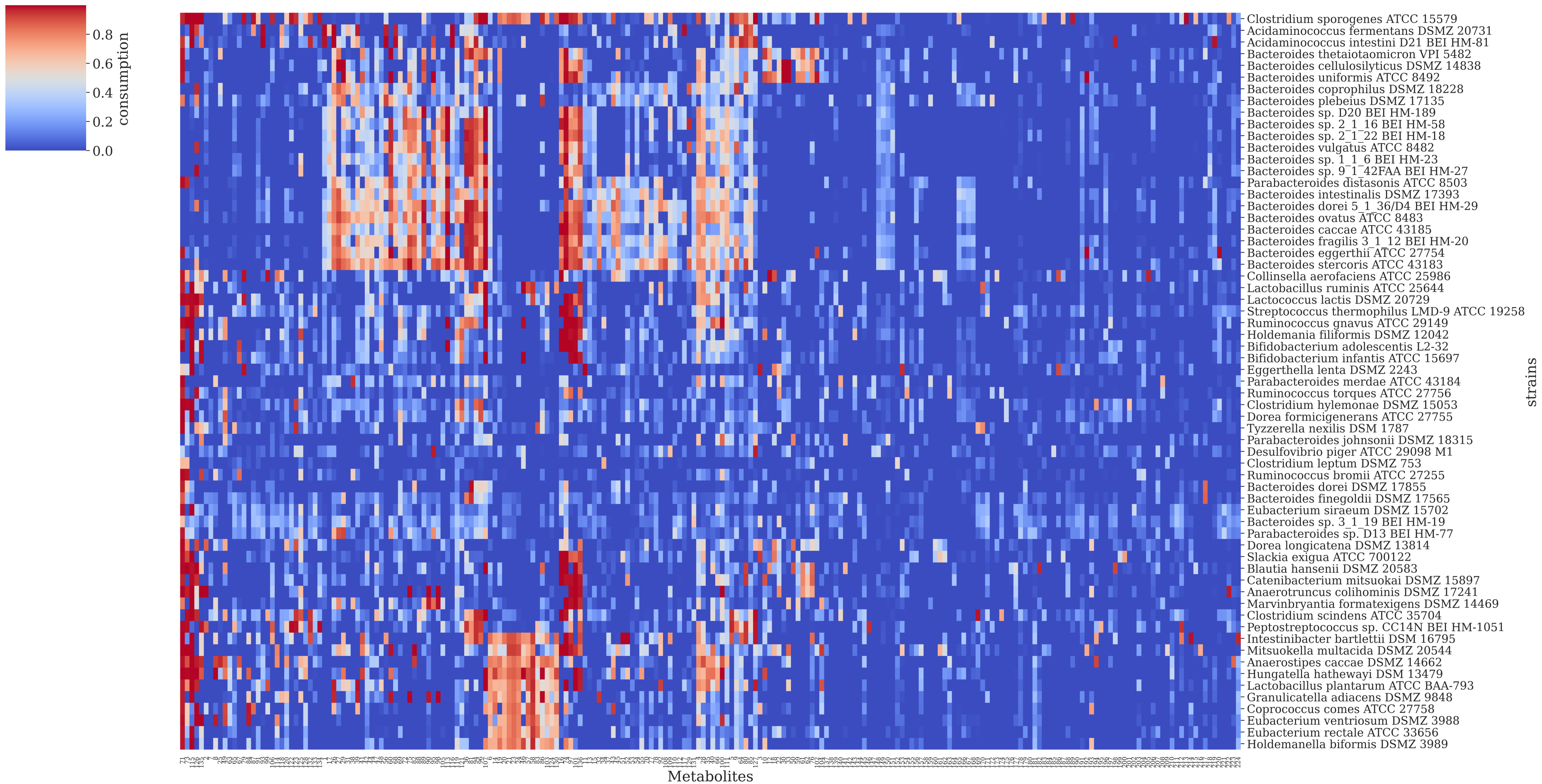

Supplement: S10 Fig — We have only included the metabolites which contributed to the biomass of at least one strain after thresholding the consumption matrix as described in the methods section. Columns and rows represent the metabolite numbers and the strains, respectively. Metabolites are clustered such that the 10 non-singleton metabolite clusters (their individual metabolites) first appear from the left, followed by the metabolites in the non-singleton clusters. The mapping between metabolite IDs on the x-axis and metabolite names are given in S3 Table. (PDF) [file pcbi.1013222.s010.pdf]

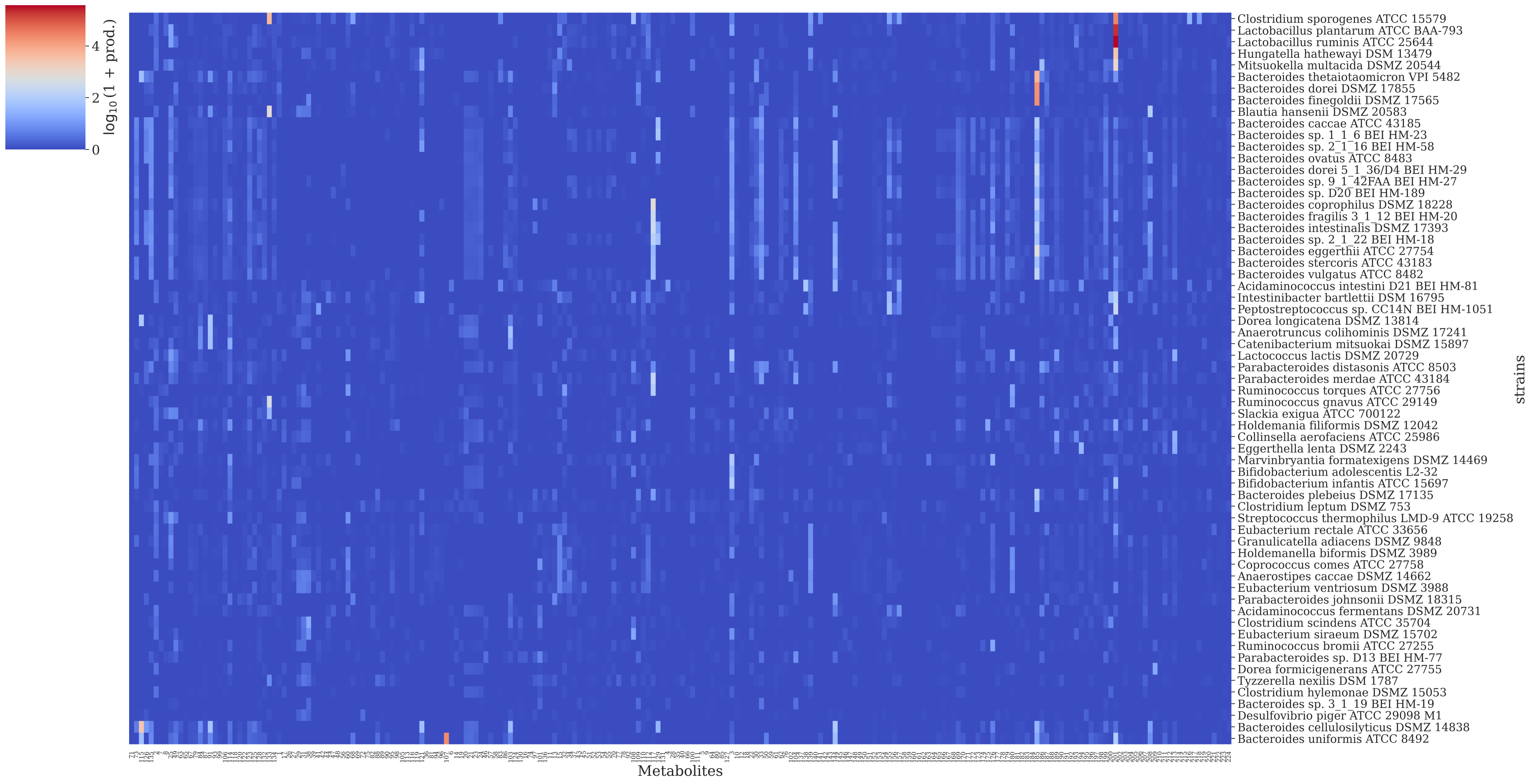

Supplement: S11 Fig — We have only included the metabolites which contributed to the biomass of at least one strain after thresholding the consumption matrix as described in the methods section. Columns and rows represent the metabolite numbers and the strains, respectively. Since the production fluxes spanned several orders of magnitude, they were log-transformed after addition of a 1 in the hearmap. Metabolites are clustered such that the 10 non-singleton metabolite clusters (their individual metabolites) first appear from the left, followed by the metabolites in the non-singleton clusters. The mapping between metabolite IDs on the x-axis and metabolite names are given in S3 Table. (PDF) [file pcbi.1013222.s011.pdf]

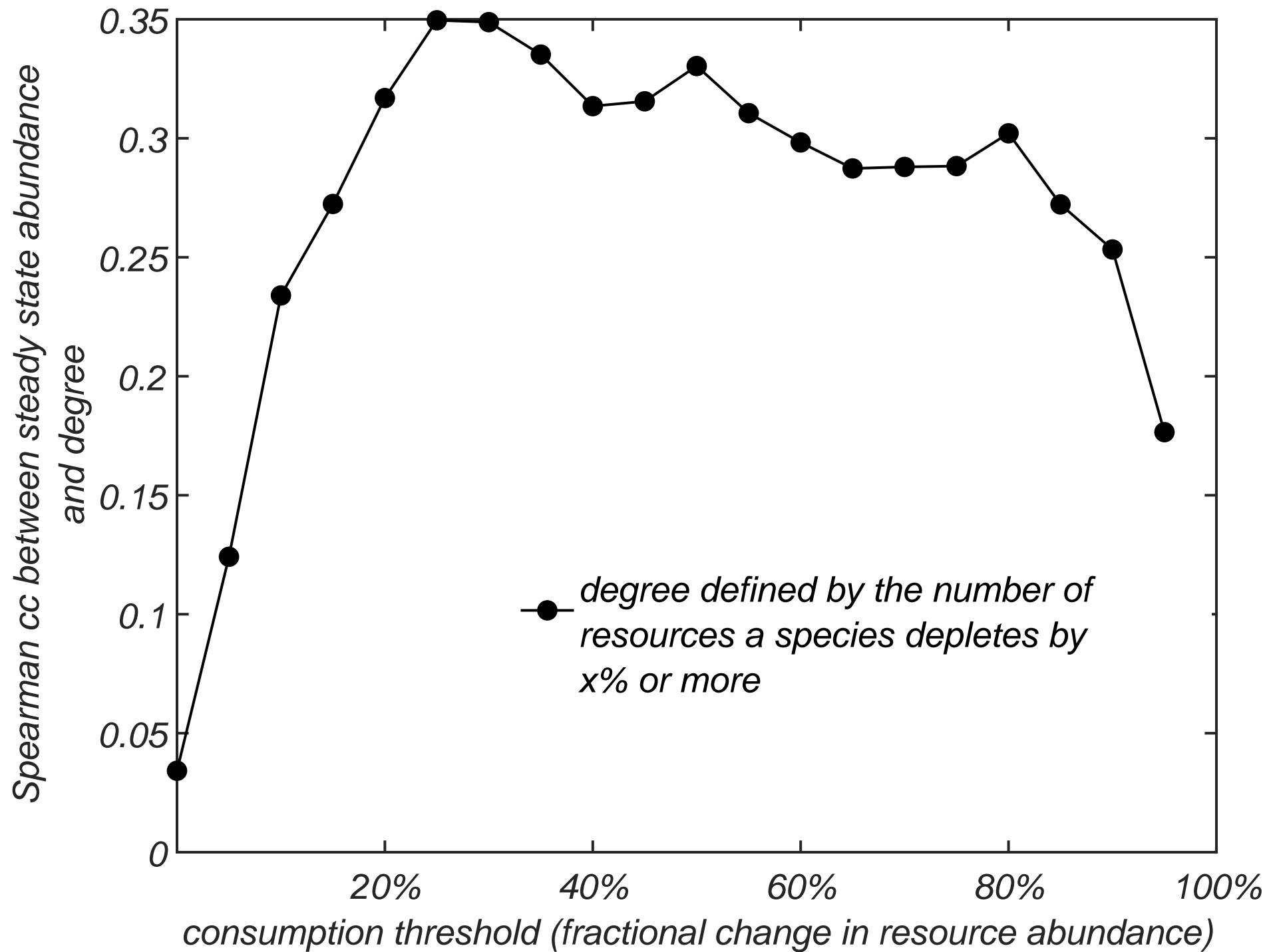

Supplement: S13 Fig — The plot illustrates Spearman’s rank correlation between steady-state strain abundances and their degree (total consumed resources) in binarized consumption fluxes, as a function of consumption threshold. A global maximum occurs at a threshold of 0.3(30%). (PDF) [file pcbi.1013222.s013.pdf]

a)

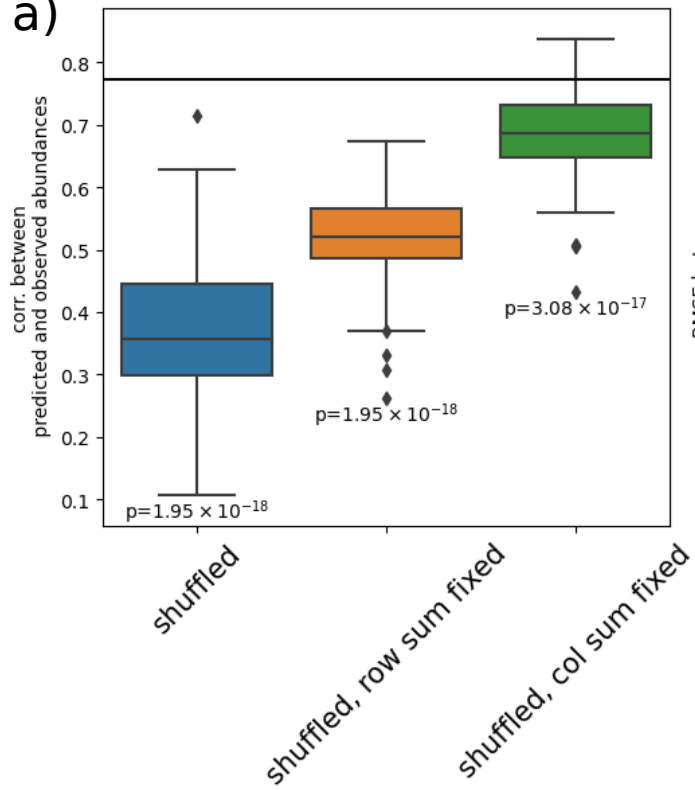

b)

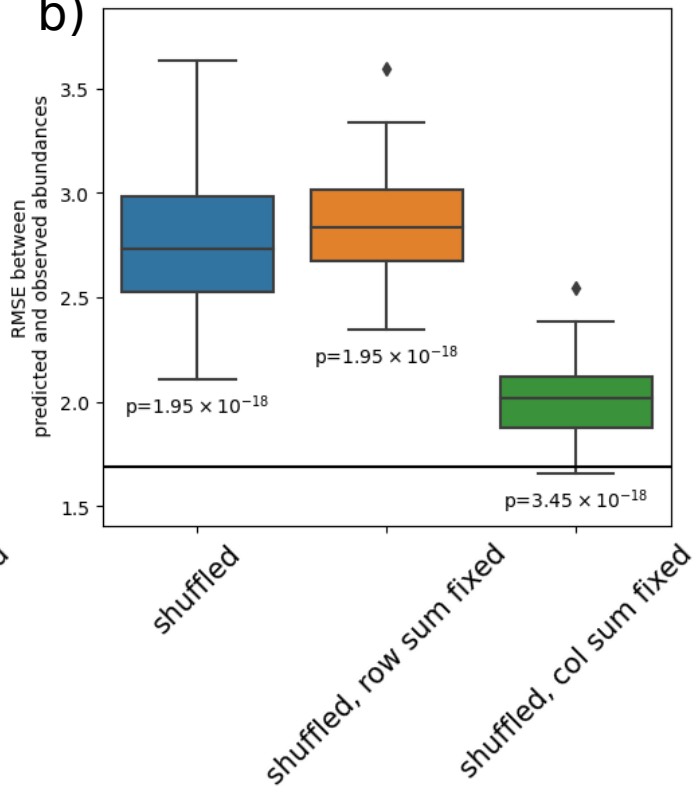

Supplement: S14 Fig — The plot depicts a) Pearson’s correlation coefficient and b) RMSE between predicted and observed steady-state (passage 3) strain abundances in the community on log10 scale. We conducted experiments with three types of shuffling of the consumption fluxes matrix (nS×nR): (1) complete randomization (shuffled), (2) row sum-preserving shuffling, and (3) column sum-preserving shuffling. The solid black line shows the performance for the model trained with the unshuffled consumption fluxes. (PDF) [file pcbi.1013222.s014.pdf]
