# Supplementary material for: Coarse-grained model of serial dilution dynamics in synthetic human gut microbiome
Source: PLoS Comput Biol. 2025 Jul 14;21(7):e1013222. doi: 10.1371/journal.pcbi.1013222 (PMC12270328; doi:10.1371/journal.pcbi.1013222)
Supplement: S4 Fig — a)–c) Cumulative log10 RMSE is plotted as a function of average observed strain abundances at steady state for model performance (blue curve) and biological replicate-to-replicate variability (red curve). First, the strains were sorted in increasing order of average observed steady-state abundances. For each value of observed steady-state abundance used as a threshold, the cumulative RMSE at each passage was calculated for a subset of strains with strain abundances at that passage greater than the threshold (details are given in the main Methods section). d)–f) The width of the error bars (maximum minus minimum) from Fig. 2 are plotted as a function of observed strain abundance for different passages. Each point on the scatterplot represents a single strain. Pearson’s correlation coefficient (cc) (along with the p-value) is given for the different passages. The linear regression fit to the scatterplot is also shown as a dashed line, with the 95% confidence interval shown as a shaded area around the linear fit. (PDF) [file pcbi.1013222.s004.pdf]

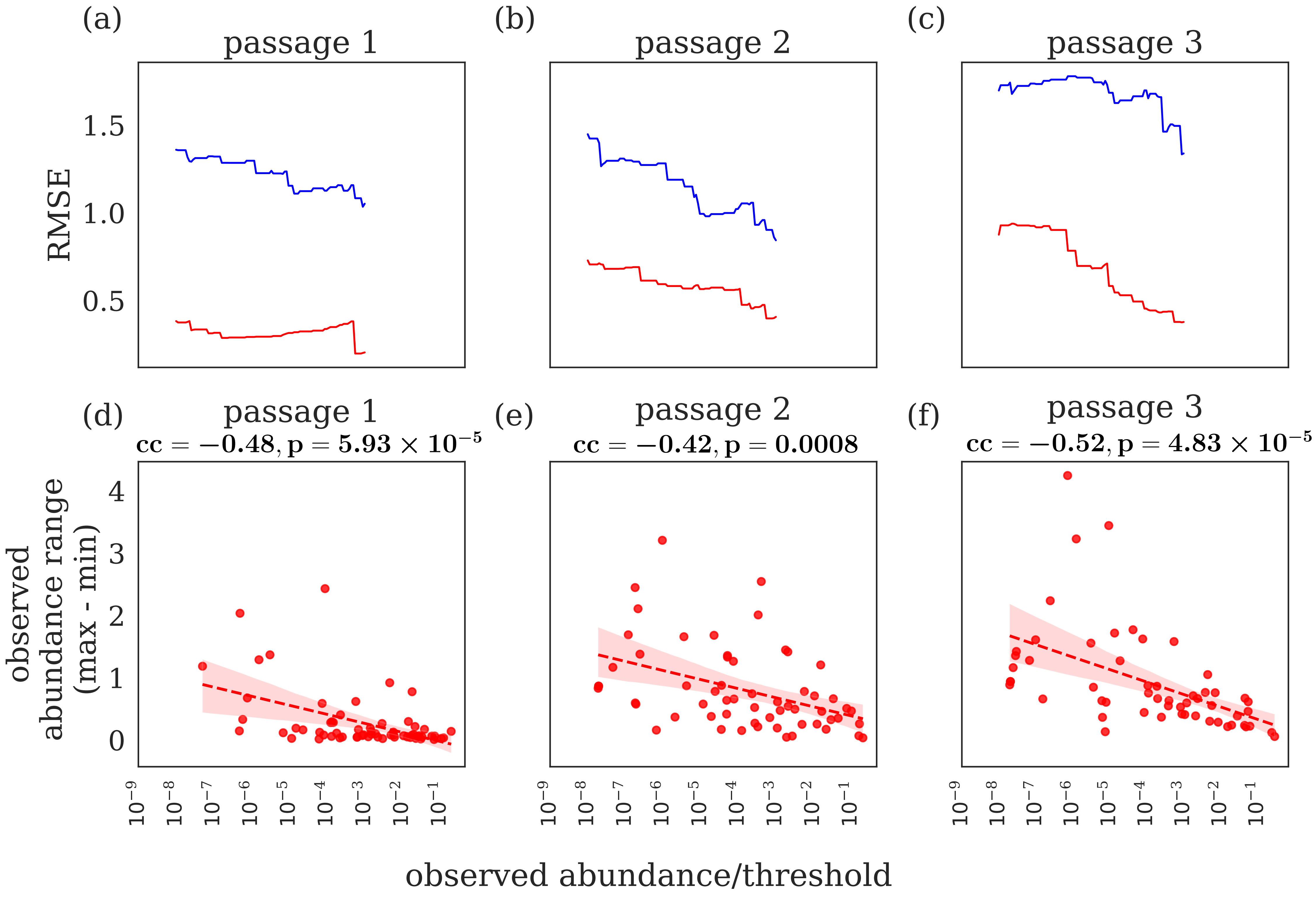
(a)
passage 1
(b)
passage 2
(c)
passage 3
RMSE
1.5
1.0
0.5
(d)
passage 1
$\mathbf{cc = -0.48, p = 5.93 \times 10^{-5}}$
(e)
passage 2
$\mathbf{cc = -0.42, p = 0.0008}$
(f)
passage 3
$\mathbf{cc = -0.52, p = 4.83 \times 10^{-5}}$
observed
abundance range
(max - min)
4
3
2
1
0
10^{-9} 10^{-8} 10^{-7} 10^{-6} 10^{-5} 10^{-4} 10^{-3} 10^{-2} 10^{-1}
observed abundance/threshold
