# Supplementary material for: Coarse-grained model of serial dilution dynamics in synthetic human gut microbiome
Source: PLoS Comput Biol. 2025 Jul 14;21(7):e1013222. doi: 10.1371/journal.pcbi.1013222 (PMC12270328; doi:10.1371/journal.pcbi.1013222)
Supplement: S6 Fig — a) Removal of the highest abundance strain from hCom2 causes steady-state abundances for multiple strains to change by different factors, which cannot be explained by re-normalization of perturbed abundances. b) Removal of a low abundance strain does not change steady-state abundances for the hCom2 community. (PDF) [file pcbi.1013222.s006.pdf]

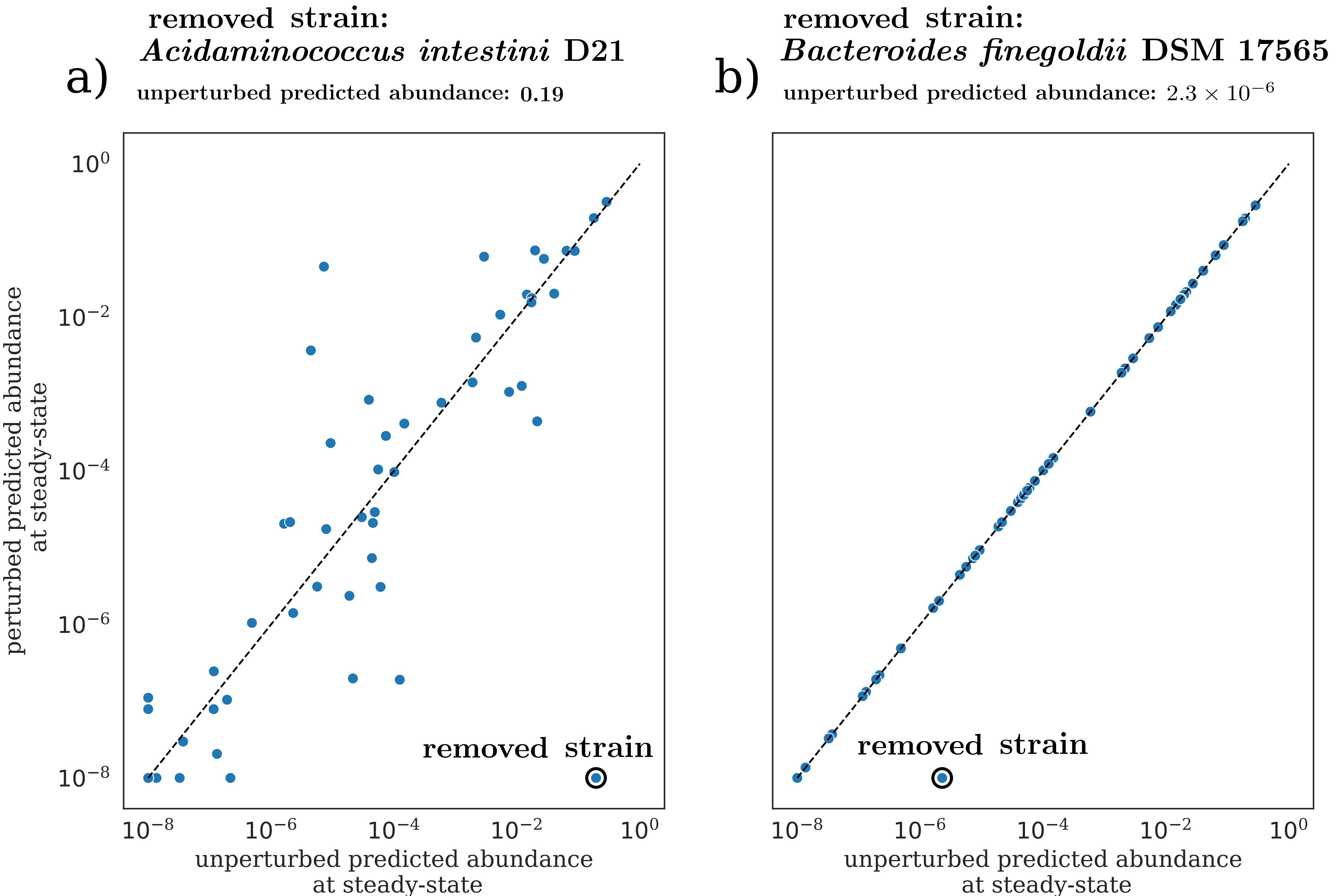
a)
removed strain:
Acidaminococcus intestini D21
unperturbed predicted abundance: 0.19
b)
removed strain:
Bacteroides finegoldii DSM 17565
unperturbed predicted abundance: 2.3×10^{-6}
perturbed predicted abundance
at steady-state
unperturbed predicted abundance
at steady-state
removed strain
10^{0}
10^{-2}
10^{-4}
10^{-6}
10^{-8}
