# Supplementary material for: Coarse-grained model of serial dilution dynamics in synthetic human gut microbiome
Source: PLoS Comput Biol. 2025 Jul 14;21(7):e1013222. doi: 10.1371/journal.pcbi.1013222 (PMC12270328; doi:10.1371/journal.pcbi.1013222)
Supplement: S12 Fig — Predictions from the monoculture null model for hCom2 plotted against observed abundances at different passages. Pearson’s correlation coefficients (cc) and p-values between log10 of predicted and observed abundances are listed above each panel. RMSE values are also shown. Each point on the scatterplot represents one strain. Error bars correspond to the range (maximum minus minimum) of observed strain abundances across three biological replicates. (PDF) [file pcbi.1013222.s012.pdf]

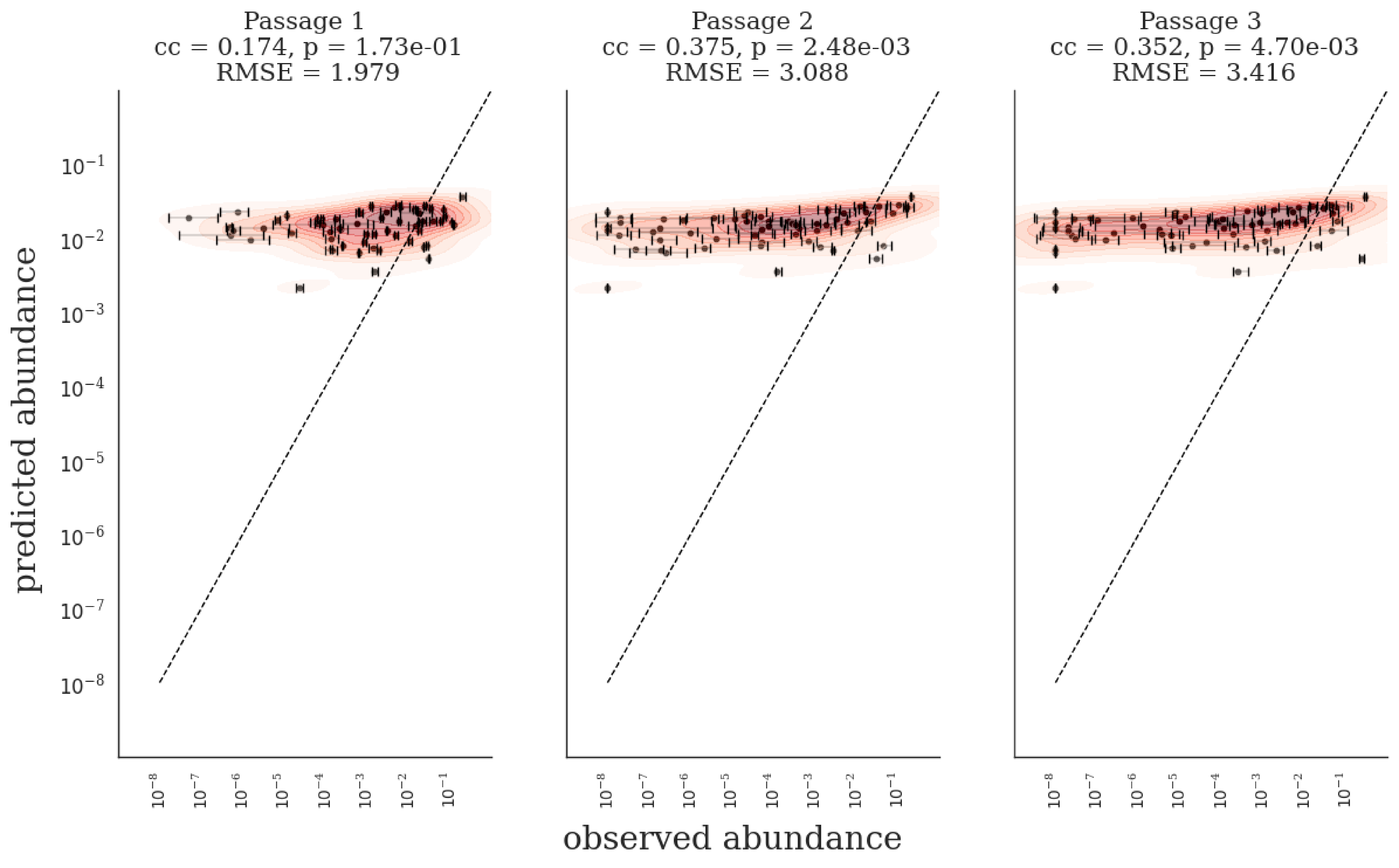
Passage 1
cc = 0.174, p = 1.73e-01
RMSE = 1.979
Passage 2
cc = 0.375, p = 2.48e-03
RMSE = 3.088
Passage 3
cc = 0.352, p = 4.70e-03
RMSE = 3.416
predicted abundance
observed abundance
10^{-1}
10^{-2}
10^{-3}
10^{-4}
10^{-5}
10^{-6}
10^{-7}
10^{-8}
