# Supplementary material for: Coarse-grained model of serial dilution dynamics in synthetic human gut microbiome
Source: PLoS Comput Biol. 2025 Jul 14;21(7):e1013222. doi: 10.1371/journal.pcbi.1013222 (PMC12270328; doi:10.1371/journal.pcbi.1013222)
Supplement: S1 Text — (PDF) [file pcbi.1013222.s015.pdf]

S1 Text. Approximate analytical estimate for time fraction f.

First, for simplicity, assume that the depletion times are similar for all the resources. Now, as stated in the main text, N_α (avg. of cycle) $= \int_0^{T_d} N_\alpha(t)/T_d$, where T_d is the common depletion time. Next, assuming approximate exponential growth with an approximately constant growth rate, g_α, we obtain

$$N_\alpha\,(\text{avg. of cycle}) = \frac{N_\alpha(T_d)}{g_\alpha T_d} = \frac{N_\alpha(T_d)}{\ln(\frac{N_\alpha(T_d)}{N_\alpha(\text{start of cycle})})} \tag{S1.1}$$

Further, assuming that the resources are consumed towards the end of the growth cycle, and $N_\alpha(T_d) \sim N_\alpha$ (end of cycle), then we obtain

$$N_\alpha\,(\text{avg. of cycle}) = \frac{N_\alpha(\text{end of cycle})}{\ln(\frac{N_\alpha(\text{end of cycle})}{N_\alpha(\text{start of cycle})})} \tag{S1.2}$$

To estimate f, we next equate the RHS of Eq (2) with the RHS of Eq (S1.2), and get

$$f = 1 - \frac{\ln(\ln(\frac{N_\alpha(\text{end of cycle})}{N_\alpha(\text{start of cycle})}))}{\ln(\frac{N_\alpha(\text{end of cycle})}{N_\alpha(\text{start of cycle})})} \sim 1 - \frac{\ln(\ln(D))}{\ln(D)}, \tag{S1.3}$$

where we have used the fact that at steady state, the ratio of N_α (end of cycle) and N_α (start of cycle) approaches the dilution factor D. In the limit $D \to \infty$, f approaches 1. For hCom2, $D = 15,0000$ and the approximate $f = 0.8$. This exercise demonstrates that for a large dilution factor, D, when resources are depleted towards the end of the growth cycle, the time fraction f is close to 1.
