# Supplementary material for: Coarse-grained model of serial dilution dynamics in synthetic human gut microbiome
Source: PLoS Comput Biol. 2025 Jul 14;21(7):e1013222. doi: 10.1371/journal.pcbi.1013222 (PMC12270328; doi:10.1371/journal.pcbi.1013222)
Supplement: S1 Table — (PDF) [file pcbi.1013222.s016.pdf]

S1 Table. Abbreviations for strain names.

Strain Abbreviation	Strain
A. fermentans	*Acidaminococcus-fermentans*-DSM-20731
A. intestini	*Acidaminococcus-intestini*-D21
A. caccae	*Anaerostipes-caccae*-DSM-14662
A. colihominis	*Anaerotruncus-colihominis*-DSM-17241
B. caccae	*Bacteroides-caccae*-ATCC-43185
B. cellulosilyticus	*Bacteroides-cellulosilyticus*-DSM-14838
B. coprophilus	*Bacteroides-coprophilus*-DSM-18228
B. dorei 5-1-36-D4	*Bacteroides-dorei*-5-1-36-D4
B. dorei DSM-17855	*Bacteroides-dorei*-DSM-17855
B. eggerthii	*Bacteroides-eggerthii*-DSM-20697
B. finegoldii	*Bacteroides-finegoldii*-DSM-17565
B. fragilis 3-1-12	*Bacteroides-fragilis*-3-1-12
B. intestinalis	*Bacteroides-intestinalis*-DSM-17393
B. ovatus	*Bacteroides-ovatus*-ATCC-8483
B. plebeius	*Bacteroides-plebeius*-DSM-17135
B. thetaiotaomicron	*Bacteroides-thetaiotaomicron*-1-1-6
B. fragilis 2-1-16	*Bacteroides-fragilis*-2-1-16
B. sp 2-1-22	*Bacteroides-sp*-2-1-22
B. sp 3-1-19	*Bacteroides-sp*-3-1-19
B. sp 9-1-42FAA	*Bacteroides-sp*-9-1-42FAA
B. sp D2	*Bacteroides-sp*-D2
B. stercoris	*Bacteroides-stercoris*-ATCC-43183
B. thetaiotaomicron	*Bacteroides-thetaiotaomicron*-VPI-5482
B. uniformis	*Bacteroides-uniformis*-ATCC-8492
B. vulgatus	*Bacteroides-vulgatus*-ATCC-8482
B. adolescentis	*Bifidobacterium-adolescentis*-L2-32
B. longum	*Bifidobacterium-longum*-ATCC-15697
B. hansenii	*Blautia-hansenii*-DSM-20583
C. mitsuokai	*Catenibacterium-mitsuokai*-DSM-15897
C. hylemonae	*Clostridium-hylemonae*-DSM-15053
C. leptum	*Clostridium-leptum*-DSM-753
C. scindens	*Clostridium-scindens*-ATCC-35704
C. sporogenes	*Clostridium-sporogenes*-ATCC-15579
C. aerofaciens	*Collinsella-aerofaciens*-ATCC-25986
C. comes	*Coprococcus-comes*-ATCC-27758
D. piger	*Desulfovibrio-piger*-ATCC-29098
D. formicigenerans	*Dorea-formicigenerans*-ATCC-27755
D. longicatena	*Dorea-longicatena*-DSM-13814
E. lenta	*Eggerthella-lenta*-DSM-2243
E. rectale	*Eubacterium-rectale*-ATCC-33656
E. siraeum	*Eubacterium-siraeum*-DSM-15702
E. ventriosum	*Eubacterium-ventriosum*-ATCC-27560
G. adiacens	*Granulicatella-adiacens*-ATCC-49175
H. biformis	*Holdemanella-biformis*-DSM-3989
H. filiformis	*Holdemania-filiformis*-DSM-12042
H. hathewayi	*Hungatella-hathewayi*-DSM-13479
I. bartlettii	*Intestinibacter-bartlettii*-DSM-16795
L. plantarum	*Lactobacillus-plantarum*-ATCC-BAA-793
L. ruminis	*Lactobacillus-ruminis*-ATCC-25644
L. lactis	*Lactococcus-lactis*-DSMZ-20729
M. formatexigens	*Marvinbryantia-formatexigens*-DSM-14469
M. multacida	*Mitsuokella-multacida*-DSM-20544
P. distasonis	*Parabacteroides-distasonis*-ATCC-8503
P. johnsonii	*Parabacteroides-johnsonii*-DSM-18315
P. merdae	*Parabacteroides-merdae*-ATCC-43184
P. sp D13	*Parabacteroides-sp*-D13
P. sp CC14N-HM-1051	*Peptostreptococcus-sp*-CC14N-HM-1051
R. bromii	*Ruminococcus-bromii*-ATCC-27255

Continued on next page

continued from previous page

**Strain Abbreviation**	**Strain**
R. gnavus	*Ruminococcus-gnavus*-ATCC-29149
R. torques	*Ruminococcus-torques*-ATCC-27756
S. exigua	*Slackia-exigua*-ATCC-700122
S. thermophilus	*Streptococcus-thermophilus*-LMD-9
T. nexilis	*Tyzzerella-nexilis*-DSM-1787
