# Supplementary material for: Coarse-grained model of serial dilution dynamics in synthetic human gut microbiome
Source: PLoS Comput Biol. 2025 Jul 14;21(7):e1013222. doi: 10.1371/journal.pcbi.1013222 (PMC12270328; doi:10.1371/journal.pcbi.1013222)
Supplement: S2 Table — (PDF) [file pcbi.1013222.s017.pdf]

S2 Table. Metabolite clusters sorted in decreasing order of R_i for hCom2.

Cluster ID	# Metabolites	Metabolites	R_i
93	1	PYROGLUTAMIC ACID	0.0897
8	7	MANNOSE 6-PHOSPHATE, GLUCOSE, SUCROSE_1, GLYCERIC ACID, 1,6-ANHYDRO-B-GLUCOSE, HYPOXANTHINE, N-ACETYLNEURAMINATE	0.0644
38	1	BETA-LACTOSE	0.0446
3	30	N-METHYL-ASPARTIC ACID_0, STEARIC ACID, CITRIC ACID, CITRAMALIC ACID, ITACONIC ACID, ACONITIC ACID, DIACETYL, PYRUVIC ACID, 4-METHYLVALERIC ACID, 2,4-DIHYDROXYBUTANOIC ACID, 5-HYDROXYMETHYL-2-FURANCARBOXYLIC ACID, N-ACETYL-CYSTEINE, GLUCONIC ACID, N-METHYL-ASPARTIC ACID_1, 2'-DEOXYGUANOSINE 5'-MONOPHOSPHATE, 4-AMINOBUTANOIC ACID, 4-ETHYLOCTANOIC ACID, ACETYL-CARNITINE, ADENOSINE 2',3'-CYCLIC MONOPHOSPHATE, ASPARTIC ACID, CYSTEINE, CYTIDINE, CYTIDINE 2',3'-CYCLIC MONOPHOSPHATE, GLYCEROL 2-PHOSPHATE, GUANOSINE 3',5'-CYCLIC MONOPHOSPHATE, RAFFINOSE, N-METHYLNICOTINAMIDE, O-ACETYL-SERINE, PALMITIC ACID, PENTANEDECANOIC ACID	0.0425
5	15	BETA-ALANINE_0, OMEGA-HYDROXYDODECANOIC ACID, IS_PHENYLALANINE-2,3,4,5,6-D5_0, IS_D15-OCTANOIC ACID_0, IS_D19-DECANOIC ACID_0, IS_TRIDECANOIC ACID_0, IS_D27-TETRADECANOIC ACID_0, XANTHINE, 4-ACETAMIDOPHENOL, 3-HYDROXYPHENYLACETIC ACID, CADAVERINE, CHOLINE, LUMICHROME, PYRIDOXAMINE, SUBERIC ACID	0.0358
89	1	PTERIN	0.0229
20	1	CYSTINE	0.0223
11	1	GALACTOSAMINE	0.0191
16	1	2-HYDROXY-2-METHYLBUTYRIC ACID	0.0190
25	1	IS_PHENYLALANINE-2,3,4,5,6-D5_1	0.0128
91	1	PYRIDOXINE	0.0128
52	1	IS_4-BROMO-PHENYLALANINE_2	0.0123
2	25	NICOTINIC ACID, 1-METHYL-HYDANTOIN, SUCCINIC ACID, LACTIC ACID, LINOLEIC ACID, IS_PROGESTERONE-D9_1, 1-METHYLGUANIDINE, 2-AMINOBENZOIC ACID, ARGININE, CAPRYLOYL GLYCINE, CYSTATHIONINE, DETHIOBIOTIN, GLUTAMIC ACID, HIPPURIC ACID, METHIONINE, N6-(DELTA2-ISOPENTENYL)-ADENINE, OPHTHALMIC ACID, PANTOTHENIC ACID, PHENYLALANINE, PROLINE, RIBOFLAVIN, SPERMINE, THREONINE_1, TRYPTAMINE, TYROSINE	0.0122
26	1	IS_4-BROMO-PHENYLALANINE_0	0.0082
33	1	MALEAMIC ACID	0.0078
37	1	DECANOIC ACID	0.0070
27	1	IS_INDOLE-2,4,5,6,7-D5-3-ACETIC ACID_0	0.0064
67	1	CYTOSINE	0.0063
81	1	N-ACETYLTRYPTOPHAN	0.0060
39	1	IS_LEUCINE-5,5,5-D3_1	0.0051
17	1	N-BENZYLFORMAMIDE	0.0043
53	1	2-METHYLBUTYRYLGLYCINE	0.0043
83	1	N-BUTYRYLGLYCINE	0.0039
54	1	3'-CMP	0.0027
58	1	5-AMINOIMIDAZOLE-4-CARBOXAMIDE-1-BETA-RIBOFURANOSIDE	0.0027

Continued on next page

continued from previous page

Cluster ID	# Metabolites	Metabolites	R_i
55	1	4-ACETAMIDOBUTANOIC ACID	0.0023
65	1	CREATININE	0.0018
46	1	ACETYLCHOLINE	0.0011
71	1	HISTAMINE	9.2×10^{-4}
43	1	GLYCINE	6.6×10^{-4}
49	1	VITAMIN D3	4.3×10^{-4}
92	1	PYROGALLOL	4×10^{-4}
40	1	IS_N-BENZOYL-D5-GLYCINE_1	2.8×10^{-4}
73	1	INDOLE-3-ETHANOL	2×10^{-4}
34	1	NORVALINE	8.4×10^{-5}
64	1	CREATINE	3.3×10^{-5}
82	1	N-ALPHA-ACETYL-ASPARAGINE	9.5×10^{-6}
60	1	AGMATINE	6.5×10^{-6}
69	1	ETHYL 4-AMINOBENZOIC ACID	0
68	1	DEOXYCARNITINE	0
70	1	N-FORMYL-METHIONINE	0
63	1	CITRULLINE	0
66	1	CYTIDINE 5'-DIPHOSPHOCHOLINE	0
78	1	N-ACETYL-LEUCINE	0
74	1	INDOLEACETIC ACID	0
96	1	TRYPTOPHAN	0
95	1	TRANEXAMIC ACID	0
94	1	SN-GLYCERO-3-PHOSPHOCHOLINE	0
90	1	PUTRESCINE	0
88	1	PHENYLACETYL-GLUTAMINE	0
87	1	ORNITHINE	0
72	1	HISTIDINE	0
86	1	LEUCINE	0
84	1	N-METHYL-GLUTAMATE	0
80	1	N-ACETYLPUTRESCINE	0
79	1	N-ACETYLMURAMIC ACID	0
77	1	N-ACETYL-ALANINE	0
76	1	N-ACETYL-5-HYDROXYTRYPTAMINE	0
75	1	INDOLELACTIC ACID	0
85	1	NEPSILON,NEPSILON,NEPSILON-TRIMETHYLLYSINE	0
62	1	BETAINE	0
1	5	5'-METHYLTHIOADENOSINE, ADENINE, NICOTINAMIDE, S-(5'-ADENOSYL)-L-HOMOCYSTEINE, URACIL	0
59	1	5'-DEOXYADENOSINE	0
23	1	IS_2-FLUROPHENYLGLYCINE_0	0
22	1	IS_CARNITINE-TRIMETHYL-D9_0	0
21	1	HEPTADECANOIC ACID	0
19	1	8-HYDROXYQUINOLINE_0	0
18	1	UROCANIC ACID	0
15	1	5,6-DIHYDROURACIL	0
24	1	IS_METHIONINE-METHYL-D3_0	0
14	1	3-HYDROXYPROPIONIC ACID	0
12	1	2-AMINO-2-METHYLPROPANOIC ACID	0
10	14	OLEIC ACID, SPERMIDINE, AMINO(4-HYDROXYCYCLOHEXYL)ACETIC ACID, 1,2-DIDECANOYL-SN-GLYCERO-3-PHOSPHOCHOLINE, IS_DI-N-OCTYL PHTHALATE-3,4,5,6-D4, METHYL-MALONIC ACID_0, MALEIC ACID, PETROSELINIC ACID, THIAMINE, IS_2-FLUROPHENYLGLYCINE_1, IS_METHIONINE-METHYL-D3_1, ISOLEUCINE, 2,6-DIAMINOHEPTANEDIOIC ACID, MALIC ACID	0

Continued on next page

continued from previous page

**Cluster ID**	**# Metabolites**	**Metabolites**	R_i
9	6	THREONINE_0, CINNAMALDEHYDE, 1-AMINOCYCLOPROPANE-1-CARBOXYLIC ACID, ASP-PHE, CARNOSINE, SERINE	0
7	24	CARNITINE, PHOSPHOCHOLINE, TRYPTOPHAN ETHYL ESTER, ADENOSINE 5'-MONOPHOSPHATE, 8-HYDROXYQUINOLINE_1, THIOACETIC ACID, 4-HYDROXYBENZALDEHYDE, CAPRYLIC ACID, IS_TRYPTOPHAN-2,4,5,6,7-D5_1, IS_INDOLE-2,4,5,6,7-D5-3-ACETIC ACID_1, IS_D19-DECANOIC ACID_1, IS_D27-TETRADECANOIC ACID_1, IS_PHENYLALANINE-2,3,4,5,6-D5_2, 5-HYDROXY-TRYPTOPHAN, ALPHA-AMINOADIPIC ACID, ALPHA-N-ACETYL-GLUTAMINE, MANNITOL, N-ACETYL-ASPARTIC ACID, N-ACETYL-GALACTOSAMINE, N-ACETYL-GLUTAMIC ACID, N-ALPHA-ACETYL-LYSINE, O-PHOSPHO-SERINE, SPHINGANINE, TAURINE	0
6	5	1-METHYLADENOSINE, ADENOSINE, GUANOSINE, INOSINE, URIDINE	0
4	5	3-UREIDOPROPIONATE, ASPARAGINE, GLUTAMINE, GLYCYL-GLYCINE, METHYLMALONIC ACID_1	0
13	1	VALINE_0	0
28	1	IS_PROGESTERONE-D9_0	0
29	1	3-HYDROXYBUTYRIC ACID	0
30	1	SARCOSINE	0
57	1	4-GUANIDINOBUTANOIC ACID	0
56	1	4-AMINOBENZOIC ACID	0
51	1	IS_LEUCINE-5,5,5-D3_2	0
50	1	BILIVERDIN	0
97	1	TYRAMINE	0
48	1	LINOLENIC ACID	0
47	1	GLUCOSAMINE	0
45	1	PHENYLETHANOLAMINE	0
44	1	ALANINE	0
42	1	IS_D15-OCTANOIC ACID_1	0
41	1	IS_4-CHLORO-PHENYLALANINE_1	0
36	1	2-DEOXY-GLUCOSE	0
35	1	ARABINOSE	0
32	1	3-FUROIC ACID	0
31	1	N,N-DIMETHYLGLYCINE_0	0
61	1	HOMOSERINE	0
98	1	VALINE_1	0
